# A framework for implementation of AI in clinical dementia care

**DOI:** 10.1002/alz70858_100317

**Published:** 2025-12-24

**Authors:** Ipsit V Vahia

**Affiliations:** ^1^ Harvard Medical School, Belmont, MA, USA; McLean Hospital, Belmont, MA, USA

## Abstract

**Background:**

With artificial intelligence (AI)‐based tools becoming more available, critical new questions arising around how these tools should be incorporated into clinical practice. This presentation will incorporate new data from ongoing studies at the Technology and Aging Lab at McLean Hospital and focus on practical challenges that must be addressed to create efficient workflows and guide regulation on using AI in clinical care

**Method:**

The Technology and Aging lab at McLean Hospital oversees a portfolio of AI‐related research projects, funded by a range of agencies including the National Institute on Aging and the National Institute of Mental Health. Based on logistical, technical, pragmatic and ethical questions encountered during these projects, we have developed a framework of key considerations when translating these tools into direct patient care. This framework now guides the use of AI‐based tools at the Division of Geriatric Psychiatry at McLean Hospital.

**Result:**

Foundational considerations around incorporating AI into clinical practice include compatibility with electronic medical records, reducing the time required for the clinician to process AI–generated data and transparency around data ownership and security. For patients, aligning an AI tool with technologies that they already own and are proficient in operating, is a key consideration. Finally, educating both clinicians and patients around optimized use of AI tools is essential. Clinicians must develop proficiency in tailoring how these tools may be deployed to an individual patient's clinical condition, availability of resources and digital literacy.

**Conclusion:**

Our experience and framework demonstrate that a “one size fits all” approach to incorporating AI into clinical care does not optimize the benefits of these tools. Personalized deployment is currently necessary, though with growing evidence and improved cost effectiveness and ease of use of these tools, they may become the default standard of care.

